# Rituximab Treatment in Lupus Nephritis Resistant to Conventional Therapy: A Single-Centre Experience

**DOI:** 10.31138/mjr.250124.rtl

**Published:** 2025-01-23

**Authors:** Esra F. Senturk, Hande Ogun, Ibrahim Durucan, Berna Yurttas, Serdal Ugurlu

**Affiliations:** 1 Division of Rheumatology, Department of Internal Medicine, Cerrahpasa Medical Faculty, Istanbul University-Cerrahpasa, Istanbul, Turkey;; 2 Division of Rheumatology, Department of Internal Medicine, Tekirdag Ismail Fehmi Cumalioglu City Hospital, Tekirdag, Turkey

**Keywords:** systemic lupus erythematosus, lupus nephritis, rituximab

## Abstract

**Background:**

Treatment-resistant lupus nephritis (LN) is a challenging condition, often causing significant morbidity. While midterm outcomes of anti-CD20 therapies are well-studied, their long-term effects remain unclear.

**Objective:**

To assess Rituximab’s long-term efficacy and safety in patients with refractory LN.

**Methods:**

The study, conducted retrospectively at a single centre between 2010–2022, included lupus nephritis patients who met the American College of Rheumatology’s criteria for SLE. We evaluated 24-hour proteinuria, creatinine clearance, serum creatinine, and SLEDAI-2K, before and after rituximab treatment. The primary endpoints as a response to treatment were defined as the attainment of a prednisolone dose of 5mg and 24-hour proteinuria of 500mg. Additionally, we investigated any adverse effects of Rituximab.

**Results:**

Patients (34 females, 13 males; mean age 42.3 years; mean disease duration 10.4 years) received a median 3 of rituximab courses. Before treatment, median amount of proteinuria was 3050mg/day. Following the final course of rituximab, the median amount of proteinuria significantly decreased to 747mg/day (p<0.001). SLEDAI-2K score also reduced significantly, from 16.3±6.2 to 7.2±4.8 (p<0.001). The mean steroid dose in the final rituximab course was 7.5±5.8 mg/day (p<0.001), compared to the initial dose of 24.13±18.47mg/day. At primary endpoint, 53.1% of patients achieved a prednisolone dose of 5mg, 36.1% had a 24-hour proteinuria level of 500mg, and 25.5% met both criteria.

**Conclusion:**

Rituximab significantly improved disease activity in lupus nephritis patients, reducing proteinuria, SLEDAI-2K scores, and allowing for steroid dose reduction. Given its efficacy and safety, rituximab may be a promising option for patients resistant to conventional therapy.

## INTRODUCTION

Systemic Lupus Erythematosus (SLE) is an autoimmune disease marked by multisystemic manifestations and a heterogeneous clinical presentation. Lupus Nephritis (LN) develops early in the disease course in more than half of patients, with renal impairment often progressing to end-stage kidney disease (ESKD) in 10–20% of cases.^1^ ESKD significantly contributes to mortality in SLE, with affected patients facing a 26-fold increased risk of all-cause mortality. Furthermore, relapses of renal disease are linked to the lower survival rates.^2^ Therefore, timely and appropriate therapy is crucial. For this purpose, conventional immunosuppressant agents are currently utilised in LN. According to recently updated recommendations, glucocorticoids combined with either mycophenolate mofetil (MMF) or cyclophosphamide (CYC) are the first choice as an initial therapy plan in LN.^3^ MMF and CYC are considered equally effective, as a meta-analysis found no significant difference in their efficacy.^4^ The Aspreva Lupus Management Study (ALMS) trial also demonstrated that LN patients treated with either CYC or MMF exhibited similar clinical improvements, with treatment response rates of 53% and 56%, respectively.^5^ However, despite these treatment options, the goals of SLE nephritis treatment have not been fully met yet, prompting the search for new agents with better response rates. Given that B-cell dysregulation is a key mechanism in the pathogenesis of the disease, B-cell depletion therapy is a viable treatment option.^6^ Scientific evidence indicates that B-cells play a significant role in immune-mediated responses, have the potential to infiltrate the tubulointerstitial space, and are essential for the formation of immune complex deposits in glomeruli.^7^ As a result, B-cell depletion therapies are increasingly popular as a next-stage strategy in treating LN. Several studies have been published to evaluate the potency and safety profile of B-cell depletion therapies in SLE and LN patients. The Exploratory Phase II/III SLE Evaluation of Rituximab (EXPLORER) trial was conducted to assess the efficacy of rituximab (RTX), an anti-CD-20 monoclonal antibody, in clinically active SLE patients with extra-renal involvement.^8^ In the trial, the RTX arm showed insufficient clinical progress to meet its primary and secondary endpoints versus the placebo. Despite these efforts, further comprehensive research with large cohorts is still needed to establish the effectiveness of such medications. In this article, we discuss the results of a retrospective, single-centred study with LN patients who received RTX, an anti-CD-20 monoclonal antibody. Our study, which provides a single-centre experience in which a large number of patients were evaluated, evaluated the effectiveness of RTX in the treatment of Turkish lupus nephritis patients with real-life data. Since there is no Belimumab treatment in our country, it is important to evaluate the effectiveness of RTX as another effective treatment alternative.

## MATERIALS AND METHODS

### Patients and study design

This single-centre retrospective study included SLE patients with biopsy-proven lupus nephritis (LN), who received at least one course of intravenous RTX between 2010 and 2022 due to LN refractory to standard of care according to EULAR’s definition.

All patients met the 2019 EULAR / ACR criteria for diagnosing SLE.^9^ Suspected nephritis cases underwent percutaneous kidney biopsies as recommended by ACR guidelines.^10^ The histological classification was conducted based on the criteria outlined by the International Society of Nephrology/Renal Pathology Society (ISN/RPS) standards.^11^

One treatment course consisted of two consecutive 1000 mg/day RTX infusions on the first and 15th day, with no additional RTX infusions until the sixth month. During this period, patients continued their current conventional immunosuppressive agents, including mycophenolate mofetil, azathioprine, cyclophosphamide, tacrolimus, or cyclosporine, without significant dose reduction. Patients under 18 years of age, as well as pregnant or actively breastfeeding patients, were excluded.

The study was approved by the Istanbul University-Cerrahpasa Clinical Research Ethics Committee with approval number E-83045809-604.01.01-661508. Written informed consent was obtained from all study participants. All individual participants consented to publish this material.

### Data Collection and Outcomes

Patient demographics and relevant clinical information, including the onset of symptoms, disease duration, concomitant comorbidities, and previous treatments at other centres, were collected from hospital medical records. Plasma creatinine levels (mg/dl), creatinine clearance rate (ml/min), and 24-hour total urine proteinuria (mg/day) were monitored through electronic medical records. Disease activity was assessed using the Systemic Lupus Erythematosus Disease Activity Index 2000 (SLEDAI-2K), which involved monitoring 24-hour urine protein levels, urinalysis (WBC and RBC cast), plasma complement concentration (C3 and C4), platelet count, white blood cell count, and anti-double-stranded DNA antibody titres every three months. We assessed 24-hour proteinuria, creatinine clearance, serum creatinine, SLEDAI-2K scores, and concomitant using corticosteroid doses before and after RTX therapy (**Table 2**). The study aimed to evaluate the efficacy of RTX in reducing the maintenance prednisolone dose to ≤ 5 mg/day and achieving a 24-hour urine protein level of ≤ 500 mg, following at least one course of treatment as the primary endpoint.

**Table 2.** Distribution of SLEDAI-2K scores, serum creatinine, creatinine clearance, 24-hour total proteinuria and mean prednisolone dose before and after RTX treatment.

	**Before Rituximab treatment**	**After Rituximab treatment**	**p-value**
SLEDAI-2K, mean ± SD	16.3 ± 6.2	7.2 ± 4.8	<0.001^a^
Serum creatinine, mean ± SD, mg/dl	0.94 ± 0.59	0.98 ± 0.68	0.53^a^
Creatinine clearance ± SD, ml/min	102.01 ± 43.2	109.1 ± 51.3	0.28^a^
24-hour total proteinuria, median, IQR (25–75), mg/dl	3050, (1370–5175)	747, (396–1500)	<0.001^b^
Mean prednisolone dose ± SD, mg	24.13 ± 18.47	7.5 ± 5.8	<0.001^a^

a Paired sample t-test;

b Wilcoxon signed-rank test.

### Adverse effects

During treatment, potential side effects of RTX were carefully examined. Persistent deep hypogammaglobulinemia, which means low immunoglobulin (Ig) levels (IgG concentration < 600 mg/L) persist for at least three months after rituximab treatment, or any anaphylaxis-like reactions, including respiratory compromise, hypotensive episodes, and loss of consciousness during the infusion, were considered serious side effects. If any of these adverse reactions were observed, the RTX treatment was deferred for safety concerns. Additionally, intravenous immunoglobulin (IVIG) treatment was administered to patients with severe persistent deep hypogammaglobulinemia, which means IgG concentration was below 400 mg/L.

### Data analysis

Statistical analysis was performed using IBM SPSS version 20.0 software (IBM Corporation, Armonk, NY, USA). Normally distributed values were expressed as mean ± standard deviation, while nonparametric values were reported as median (minimum, maximum levels).) The normality was assessed by analytic methods (Kolmogorov-Smirnov/Shapiro-Wilk test) and visual histogram interpretation. As the outcomes were compared before or after courses of RTX treatment in the same patient group (dependent variable), the Paired t-test was used for parametric values, and the Wilcoxon test for non-parametric values.

## RESULTS

Forty-seven (34 F, 13 M) patients with biopsy-proven SLE nephritis treated with RTX were evaluated. The baseline characteristics of patients are presented in **Table 1**. Most patients were female (64.2%), and the mean ± SD age of the patients was 42.3 ± 11.1 years. All patients had active lupus nephritis at the time of initiation. The mean disease duration was 10.44 ± 6.9 years. Histopathological classification of LN (ISN/RPS) was Class II in 5 cases (10.6%) Class III in 7 cases (14.8%), Class IV in 16 cases (34.0%), Class V in 6 cases (12.7%), Class III+V in 7 cases (14.8%), and Class IV+V in 6 cases (12.7%). The other medications the patients were previously treated with were cyclophosphamide in 35 cases (74.4%), mycophenolate mofetil in 31 cases (65.9%), azathioprine in 6 cases (12.7%), and cyclosporine in 2 cases (4.2%), and some patients had received more than one type of therapy. Extra-renal involvements are mucocutaneous in 20 cases (42.5%), joint in 18 cases (38.2%), haematological in 13 cases (27.6%), cardiopulmonary in 15 cases (31.9%), and neuropsychiatric in 2 cases (4.2%). All patients were given one of the standard treatments (azathioprine, cyclophosphamide, or mycophenolate mofetil) along with RTX. Before receiving RTX therapy, 35 out of 47 patients who were refractory to treatment were administered cyclophosphamide. The remaining twelve patients received mycophenolate mofetil therapy prior to RTX. Among those twelve patients, 7 were 40 years old or younger and chose not to receive cyclophosphamide due to concerns about potential pregnancy or infertility issues. Patients received a median of 3.00 [Minimum-Maximum (1–20)] courses of RTX (**Table 1**). As a result of this treatment, the median amount of proteinuria, which was 3050 (1370 - 5175) mg/day pre-treatment, decreased to 747 (396 - 1500) mg/day after the final course (p< 0.001). The serum creatinine levels before and after the treatment were 0.94 ± 0.59 and 0.98 ± 0.68 (p = 0.53). Mean serum creatinine clearance increased from 102.01 ± 43.2 to 109.1 ± 51.3 (p = 0.28). The mean SLEDAI-2K score at baseline was 16.3 ± 6.2 points and reduced to 7.2 ± 4.8 (p< 0.001). RTX was used in combination with oral corticosteroids. After RTX treatment, the daily corticosteroid dose decreased from 24.13 ± 18.47 mg/day to 7.5 ± 5.8 mg/day (p< 0.001). The end-stage renal disease developed in 3 (6%) patients.

**Table 1.** Baseline characteristics of the patients.

**Age, mean ± SD years**	42.3 ± 11.1

**Gender (n)**	34 (72.3%)
Female	13 (27.6%)
Male	

**Disease duration, mean ± SD years**	10.44 ± 6.9

**Histological classification (ISN/RPS Classification) (n%)**	5 (10.6%)
Class II	7 (14.8%)
Class III	16 (34.0%)
Class IV	6 (12.7%)
Class V	7 (14.8%)
Class III+V	6 (12.7%)
Class IV+V	

**RTX treatment courses median (min-max)**	3.00 (1–20)

**Previous treatments before initiation of rituximab (n%)**	
Cyclophosphamide	35 (74.4%)
Mycophenolate mofetil	31 (65.9%)
Azathioprine	6 (12.7%)
Cyclosporine	2 (4.2%)

**Extrarenal involvement, N/Total (%)**	
Mucocutaneous	20/47(42.5%)
Joint	18/47(38.2%)
Haematological	13/47(27.6%)
Cardiopulmonary	15/47 (31.9%)
Neuropsychiatric	2/47 (4.2%)

In terms of the primary endpoint, a current prednisolone dose of ≤ 5mg and 24-hour proteinuria ≤ 500 mg were achieved in 53.1% and 36.1% of patients, respectively. Both criteria were met by 25.5% of patients (**Table 3**). The percentage of patients achieving the primary endpoint for each renal pathology class is given in **Table 4**. Most patients who reached the primary endpoint had class 3 lupus nephritis (57.1%) (**Table 4**).

**Table 3.** Primary outcome measures.

**Primary endpoint**	**Number of patients (N=47)**
Current prednisolone dose ≤5 mg, (n, %)	25 (53.1%)
24-hour proteinuria ≤ 500mg, (n, %)	17 (36.1%)
Current prednisolone dose ≤5 mg and 24-hour proteinuria ≤ 500 mg, (n, %)	12 (25.5%)

**Table 4.** Reaching the primary endpoint for each renal pathology class.

**Class**	**Current prednisolone dose ≤5 mg and 24-hour proteinuria ≤ 500 mg, N(%)**	**P value**
II	1(20.0%)	0.59*
III	4 (57.1%)	
III +V	0 (0.0%)	
IV	5 (31.3%)	
IV+V	1(16.7%)	
V	1(16.7%)	

* Pearson Chi-square test; no significant difference was noted among any of the classes to primary endpoints, characterised by lowering the prednisolone dose of less than 5 mg and 24-hour proteinuria of less than 500 mg.

In **Table 5**, we identify the baseline characteristics of non-responders and responders to treatment. The only significant difference was the pre-treatment median proteinuria level: 1.85 g/dL for responders and 3.83 g/dL for non-responders (p = 0.04).

**Table 5.** Non-responders versus Responders baseline characteristics.

	**Responders**	**Non-responders**	**P value**
Age [median (min-max)]	41.5 (23.00–66.00)	41 (22.00–70.00)	0.55
Disease duration [Median (min-max), (years)]	7.0 (2.00–33.00)	9.0 (1.00–22.00)	0.58
Pre-treatment creatinine [Median (min-max), (mg/dl)]	0.76 (0.48–2.46)	0.72 (0.28–2.84)	0.85
Pre-treatment proteinuria [Median, (IQR), g/dl)]	1.85 (2.87)	3.83 (4.12)	0.04
Pre-treatment SLE-DAI [(Median (min-max)]	19.0 (4.00–28.00)	16.0 (0.00–27.00)	0.12

During the treatment, 2 patients had serum reactions, 3 had pneumonia, and 3 had herpes zoster. Two patients had severe persistent hypogammaglobulinemia and were treated with intravenous immunoglobulin. No one died of lupus nephritis.

## DISCUSSION

This study presents RTX as an alternative to conventional treatments for lupus nephritis. Off-label use of RTX in lupus nephritis was first introduced in the literature in 2002.^13^ Several case reports and retrospective studies show that RTX is effective in refractory lupus nephritis.^14–17^ Two randomised clinical trials, i.e., the LUNAR (Lupus Nephritis Assessment with Rituximab) and the Exploratory Phase II/III SLE Evaluation of Rituximab (EXPLORER), failed to show the effectiveness of RTX in lupus patients.^18,19^ However, our study and the LUNAR trial had different inclusion criteria. In the LUNAR group, patients were administered mycophenolate as an induction therapy before RTX infusion at the initial renal flare. In our series, most patients received cyclophosphamide and mycophenolate (75% and 65%, respectively) during their treatment period. Unlike the LUNAR trial, RTX courses were not initiated during the onset of a renal flare. The ethnic background of patients in our series was also distinct, primarily including Caucasian individuals, while the LUNAR trial primarily involved African-Americans.

A complete response was achieved in 26.4% of the RTX group and 30.6% in the placebo arm during 52 weeks in the LUNAR trial. Parallel to the LUNAR trial, we achieved the primary endpoint of only 25.5% of our series, referred to as the current prednisolone dose ≤5 mg and 24-hour proteinuria ≤ 500 mg. Most patients didn’t accomplish remission only with cyclophosphamide or mycophenolate induction treatment protocol. Thus, our study population can be considered resistant to conventional treatment. For this reason, our series may not be directly comparable with the LUNAR trial.

Class IV lupus nephritis is considered to have a poor prognosis in terms of treatment response.^5^ One main reason for not reaching the primary endpoint can be attributable to the fact that 34% and 12.7% of patients had class IV and class IV+V lupus nephritis, respectively, in our study. Most patients who reached the primary endpoint were class 3 lupus nephritis (57.1%) (**Table 4**). Like our series, two-thirds of the patients in the LUNAR trial were class IV lupus nephritis and showed a poor response.

25.5% of our patients accomplished the primary endpoint, and 36.1% had 500mg < proteinuria at the end. The median proteinuria level was reduced from 3050 (1370, 5175) mg/day to 747 (396,1500) mg/day in our series, which may, at first glance, appear unsatisfactory in achieving the primary endpoint. However, this result can be interpreted as valuable because the marked reduction in urine protein level can also prevent rapid progression to end-stage renal disease. Unlike the urine protein level, the plasma creatinine level did not change significantly with the courses of RTX treatment, reflecting that the glomerular functions were not worsened. Although this outcome was not significant, it implies that progressive reduction in glomerular function is precluded to some extent with the course of RTX.

SLE-DAI-2K score is an accepted method to validate disease activity. We compared the SLE-DAI-2K score before and after the RTX treatment. The mean SLEDAI-2K score at baseline was 16.3 ± 6.2 points and decreased to 7.2 ± 4.8 (p< 0.001). This outcome is consistent with a recently published meta-analysis and a retrospective study investigating the use of RTX for treating lupus nephritis.^20,21^ These studies indicate that concomitant RTX courses lead to improved disease activity scores.

B-cell depletion is the main mechanism of RTX, and a study showed that at the beginning of treatment, checking the B-cell plasma count is an early predictor of renal response.^21^ In the non-responder group, failure of early B cell depletion was identified as a potential cause of treatment failure, which was shown to be high in SLE patients in a few studies.^22,23^ However, the blood B-cell count was not consistently assessed in our series until it was required for rare patients.^15,24^

One main advantage of our study was that we applied the same RTX treatment to the patients, leading to homogeneity in our series, as opposed to contemporary meta-analyses, which consist of studies with different RTX protocols, leading to heterogeneity in the data.^25,26^ Another advantage of our study is the follow-up time. The majority of the patients received a minimum of four RTX courses, allowing at least two years for follow-up. In contrast, most studies in the literature have shorter follow-up periods of 6–12 months.^25, 27,28^

RTX has a relative safety profile in our series, which is consistent with the meta-analysis and randomised controlled trials.^18,19,25^ Two patients had serum reactions that were not life-threatening, 3 had pneumonia, and 3 had herpes zoster during the treatment. Two patients had severe hypogammaglobulinemia and were treated with intravenous immunoglobulin. Although there were no serious infectious diseases, immunoglobulin treatment costs caused a burden on the healthcare system.

## LIMITATIONS

Our study has several limitations. One major limitation of our study is that the potential depletion of B cell count was not documented after RTX treatment, which is the anticipated outcome of anti-CD20 inhibition. Treatment refractory cases can be associated with inadequate B-cell reduction in our series. Another major limitation is the retrospective design of our series, which is established by collecting old medical records for the past ten years. However, our study was conducted in a single experienced institution with regular patient follow-ups that resulted in a homogenous distribution of our data, making it consistent and reliable. Our study evaluated the effectiveness of Rituximab in the treatment of Turkish lupus nephritis patients with real-life data. Since there is no Belimumab treatment in our country, it is important to evaluate the effectiveness of Rituximab as another effective treatment alternative.

## CONCLUSION

The treatment of SLE nephritis is a challenging medical problem. Conventional therapy does not always give the expected results. Based on our clinical outcome, Rituximab can be one of the alternative agents that could have conceivable results in conventional treatment-resistant SLE nephritis. Accompanying infection risk, though increased, could be a reasonable trade-off for resistant cases, given that the vast majority of infections are not considered severe or sepsis. Nevertheless, larger prospective cohorts with longer durations are required to elucidate the risks and benefits and standardise the duration of RTX courses in SLE nephritis.

## FINANCIAL DISCLOSURE

No funding was received to conduct this study.

## CONFLICT OF INTEREST

The authors declare that they have no conflict of interest.

## DISCLAIMER

Some part of the data regarding this original article had been previously used by the same authors in “scientific abstract” format during European League Against Rheumatism (EULAR) 2023 Congress (https://doi.org/10.1136/annrheumdis-2023-eular.5000). However, research extent was optimised, and new clinical parameters were added to monitor clinical status of the patients in this original article when compared to previous “scientific abstract” publication. All authors declare that the final version of this original article has not been published in any publication source.

## COMPLIANCE WITH ETHICAL STANDARDS

This study was approved by the local ethics committee of our institution.

## DATA AVAILABILITY

The data underlying this article will be shared on reasonable request to the corresponding author**.**

## AUTHOR CONTRIBUTION

All authors contributed equally to the study conception, design, material preparation, data collection and analysis. Esra F. Senturk and Hande Ogun labelled as first author due to their efforts during research project. All authors approve the final manuscript.

## INFORMED CONSENT AND COMPLIANCE WITH ETHICAL STANDARDS

The study was approved by Istanbul University-Cerrahpasa Clinical Research Ethics Committee with approval number of E-83045809-604.01.01-661508. Written informed consent was obtained from all study participants. All individual participants consented to publication of this material.
